# The Swedish National Facility for Magnetoencephalography Parkinson’s disease dataset

**DOI:** 10.1038/s41597-024-02987-w

**Published:** 2024-01-31

**Authors:** Mikkel C. Vinding, Allison Eriksson, Igori Comarovschii, Josefine Waldthaler, Cassia Low Manting, Robert Oostenveld, Martin Ingvar, Per Svenningsson, Daniel Lundqvist

**Affiliations:** 1 NatMEG, Department of Clinical Neuroscience, Karolinska Institutet, Stockholm, Sweden; 2 Danish Research Centre for Magnetic Resonance, Centre for Functional and Diagnostic Imaging and Research, Copenhagen University Hospital - Amager and Hvidovre, Copenhagen, Denmark; 3 Department of Women’s and Children’s Health, Uppsala University, Uppsala, Sweden; 4 Department of Neurology, University Hospital Marburg, Marburg, Germany; 5 Department of Brain and Cognitive Sciences, Massachusetts Institute of Technology, Cambridge, MA 02139 USA; 6 McGovern Institute of Brain Research, Massachusetts Institute of Technology, Cambridge, MA 02139 USA; 7 Donders Institute for Brain, Cognition and Behaviour, Radboud University, Nijmegen, Netherlands; 8 Section of Neurology, Department of Clinical Neuroscience, Karolinska Institutet, Stockholm, Sweden

**Keywords:** Parkinson's disease, Sensorimotor processing

## Abstract

Parkinson’s disease (PD) is characterised by a loss of dopamine and dopaminergic cells. The consequences hereof are widespread network disturbances in brain function. It is an ongoing topic of investigation how the disease-related changes in brain function manifest in PD relate to clinical symptoms. We present The Swedish National Facility for Magnetoencephalography Parkinson’s Disease Dataset (NatMEG-PD) as an Open Science contribution to identify the functional neural signatures of Parkinson’s disease and contribute to diagnosis and treatment. The dataset contains whole-head magnetoencephalographic (MEG) recordings from 66 well-characterised PD patients on their regular dose of dopamine replacement therapy and 68 age- and sex-matched healthy controls. NatMEG-PD contains three-minute eyes-closed resting-state MEG, MEG during an active movement task, and MEG during passive movements. The data includes anonymised MRI for source analysis and clinical scores. MEG data is rich in nature and can be used to explore numerous functional features. By sharing these data, we hope other researchers will contribute to advancing our understanding of the relationship between brain activity and disease state or symptoms.

## Background & Summary

Parkinson’s disease (PD) is a common neurodegenerative disease characterised by movement symptoms, such as tremor, rigidity, and bradykinesia, and commonly accompanied by a wide range of non-motor symptoms^1,2^. The pathology of PD is characterised by dopamine depletion and loss of dopaminergic cells. The consequences hereof are widespread network disturbances throughout the brain impacting movements, behaviour, cognition, and emotion. There is still a lot to learn about the characteristics of the network level changes, how these changes manifest throughout the disease progression, as well as if and how functional changes are related to specific manifestations of disease symptoms.

We present The Swedish National Facility for Magnetoencephalography Parkinson’s Disease Dataset (NatMEG-PD) with magnetoencephalography (MEG) from human participants with PD and matched healthy controls. We hope that the NatMEG-PD can help generate new knowledge about the functional brain signatures of PD.

MEG has a high temporal resolution and great spatial coverage with high dimensionality that can be analysed in countless ways, ranging from focal time- or frequency-specific activity, in predefined regions of interest to exploratory analyses using the full range of the data^3^. This can range from extracting specific features of interest in time and/or space to using the entire dataset in machine learning analyses. NatMEG-PD is useful both as a resource for exploring basic scientific hypotheses on the role of neural activity in PD and as a resource for developing tools or algorithms to classify patients and controls or to predict specific disease symptoms.

Several features in MEG data have the potential to shed light on disease mechanisms in PD or serve as functional biomarkers of PD. For example, PD patients exhibit abnormal activity in the beta-band (~12–30 Hz) throughout the basal ganglia-cortical loops^4^, detectable at the cortical level with MEG. The changes in beta-band activity are seen both at rest^5,6^ and as a reduction in the range of typical movement-related beta-band dynamics^7,8^. The levels of beta-band activity in the sensorimotor cortex have further been linked to the severity of rigidity and bradykinesia^9^. However, the disease-related beta-band changes in PD might not be uniform across the disease, as there are reports of increased cortical beta-band power in the early stages of PD^10^, whereas later stages of PD show decreased beta-band power^11^. PD patients also show a peak shift in the oscillatory power towards lower frequencies compared to healthy controls, suggesting a “slowing down” of the brain rhythms^6,12,13^.

In both PD patients and healthy controls, the beta-band exhibits short transient “*bursts*” lasting 50–200 ms, which can be extracted from MEG signals^14^. However, the prevalence of beta bursts is reduced in PD patients^15^, and burst duration has been shown to be altered by dopaminergic medication^16^. A similar development in time-domain analysis is *waveform analysis*, a method quantifying the *sharpness* or *symmetry* of peaks in EEG signals, which has shown that deep brain stimulation in PD smoothes the sharpness of peaks in the beta band^17^. With NatMEG-PD, it will be possible to further explore similar potential functional markers of PD.

However, most evidence on oscillatory changes in PD comes from studies with small sizes and varying methodological approaches^18^. There is, therefore, a lack of established validated functional signatures of PD. This is further complicated by the fact that in addition to disease-related changes, many potential markers also show changes due to age^19,20^. There may also sex differences in the manifestation of PD^2,21^, which have not been systematically investigated in functional imaging studies. We hope that NatMEG-PD can be used for further analysis of the interaction between age, sex, and PD. The number and scope of participants included in NatMEG-PD should be comprehensive enough to carry out meaningful analyses of these factors.

The MEG data in NatMEG-PD is released as (almost) raw data in accordance with the Brain Imaging Data Structure (BIDS)^22^ in order to maximise the reusability of the data by enabling researchers to use their preferred analysis strategy and pursue their own research questions.

## Methods

### Ethical statement

The study was approved by the regional ethics committee (Etikprövningsnämden Stockholm, DNR 2019-00542) and in accordance with the Declaration of Helsinki. Participants were given written and oral information about the aims and procedures before participating in the study. All participants gave written informed consent to participate, and to having the research data shared, prior to their participation.

### Participants

NatMEG-PD includes data from 66 PD patients (age 44–77; 28 female) and 68 healthy controls (age 46–78; 30 female). Group-level summaries of the participants are presented in Table 1.

**Table 1:** Summary of the participants in NatMEG-PD.

Measure	Parkinson’s patients (N = 66)		Healthy controls (N = 68)	
Sex (female/male)	28/38	30/38		
	Mean (s.d)	Range	Mean (s.d)	Range
Age (years)	64.4 (9.8)	44–77	63.3 (8.7)	46–78
Disease duration (years)	4.0 (3.4)	0–19		
LEDD (mg)	512 (278)	0–1130		
MDS-UPDRS-III	19.3 (11.1)	1–47		
MoCA	26.5 (2.7)	18–30	26.1 (2.3)	20–30
FAB	16.3 (1.7)	12–18	17.0 (1.3)	12–18
BDI-II	7.9 (5.4)	0–22	3.4 (4.0)	0–16

PD patients were recruited from the Parkinson’s Outpatient Clinic, Department of Neurology, Karolinska University Hospital, Stockholm, Sweden. Patients were checked for eligibility according to the inclusion/exclusion criteria by a neurologist specialised in movement disorders before being scheduled for the MEG experiment. The healthy controls were recruited by advertising in local newspapers or amongst spouses of PD patients who had enrolled in the study.

The inclusion criteria for the PD group were a diagnosis of PD according to the United Kingdom Parkinson’s Disease Society Brain Bank Diagnostic Criteria with Hoehn and Yahr stage ≤3^23^. The PD patients participated in the study while on their regular prescribed dose of medication (documented in the dataset, see below).

Exclusion criteria for both groups were a diagnosis of major depression, dementia, history or presence of schizophrenia, bipolar disorder, epilepsy, or history of alcoholism or drug addiction according to the *Diagnostic and Statistical Manual of Mental Disorders*^24^, and any metal in the head or upper body. Additional exclusion criteria for the control group were a diagnosis of PD, any form of movement disorder, or a history of neurological disorders.

### MEG data acquisition

MEG data were recorded with a Neuromag TRIUX 306-channel MEG system, with 102 magnetometers and 102 pairs of planar gradiometers. Data were digitalised at 1000 Hz with an online 0.1 Hz high-pass filter and 330 Hz low-pass filter. The MEG scanner was located inside a two-layer magnetically shielded room (MSR; Vacuumschmelze GmbH) with internal active shielding to suppress electromagnetic artefacts. The MEG data were obtained in a single session that comprised several experimental tasks.

The participants’ head position and head movements inside the MEG scanner were measured during recordings with head-position indicator coils (HPI) attached to the subjects’ heads. The HPI location, nasion-, left-, and right pre-auricular points were digitalised with a Polhemus Fastrak motion tracker before the measurements, with about 100–200 additional head shape points sampled uniformly across the scalp. Horizontal and vertical electrooculogram (EOG), electrocardiogram (ECG), and electromyogram (EMG) from the right forearm appproximatly above the flexor carpi radialis were recorded simultaneously with the MEG. An accelerometer attached to the nail of the right index finger measured finger movements along three orthogonal axes and was sampled together with the MEG data.

Two minutes of empty room recordings were obtained for each participant while the participant was being prepared for the MEG recording session.

### Experimental design and procedure

**Fig. 1:**
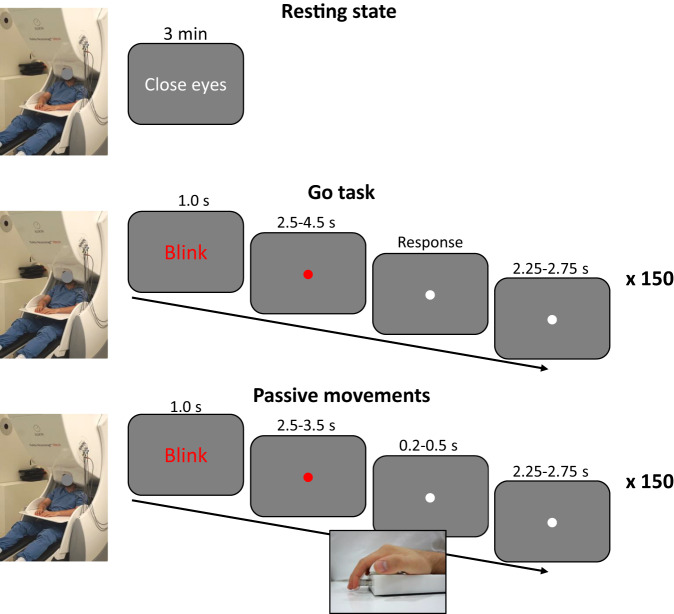
Overview of the MEG tasks. Top: three minutes resting state with the eyes closed. Middle: go task; participants had to respond when a visual cue changed colour after a random delay of 2.5-4.5 s. Bottom: passive movements; a device would induce passive movements after a random delay of 2.5-3.5 s. The visual stimuli during the passive movements were matched to the go task.

All MEG recordings were done with the participants sitting in the upright position facing a screen with images projected from outside the MSR. The following three tasks were performed (Fig. 1):

#### Resting state

We recorded three minutes of resting-state MEG while the participants sat with their eyes closed. The participants were instructed to close their eyes, sit still, and relax. The recordings began after assuring the participant sat still with their eyes closed. The start and stop of the resting-state period are marked in the trigger channel (*STI101*) in the MEG data file.

#### Go task

The participants were instructed to respond to a visual cue presented on the screen by pressing a button with their right index finger. The visual cue consisted of a fixed red dot in the middle of the screen. The dot would change colour to white at a random time between 2.5–4.5 s after trial onset. The instructions were to respond as soon as they saw the dot change colour. After the participant pressed the button, there would be a delay of between 2.25–2.75 s. The onset of the visual cue and the onset of the button press are marked in the trigger channels in the MEG data (see *Usage Notes*). A screen telling the participants to blink signified that a new trial would begin. The task was repeated for 150 trials, split into six blocks of 25 trials with a short intermediate break to counter fatigue. The participants were given a few training trials to familiarise themselves with the task before the recording began. The recording began once the experimenter had ensured that the participants understood and performed the task correctly.

#### Passive movement

Proprioceptive stimulation was induced through passive movements of the right index finger by a custom-made MEG-compatible pneumatic movement actuator^25^. The visual stimulus was matched to the *go task*, except that the participants were explicitly instructed to relax, not to move at all, and focus their gaze on the visual stimuli. The dot changed colour after a random time interval of 2.5–3.5 s, upon which the passive movement would be induced after an additional interval of 0.2–0.5 s. The movement actuator would move the finger for 0.15 s giving a movement duration of about 0.3 s. The onset of the visual cue and movement actuator are marked in the trigger channels in the MEG data (see *Usage Notes*). The movement kinematics can be evaluated using the accelerometer data. There would then be a delay of between 2.25–2.75 s after the activation of the movement actuator before a new trial began. The procedure was repeated for 150 trials. The procedure would begin after the experimenter had ensured that the participant had their finger placed correctly on the movement actuator and that the participants perceived the passive movement.

The order of the tasks was as presented above and fixed for all participants. Six of the participants did not complete the last *passive movement* task. After the resting state and movement tasks, the participants would continue with either an antisaccade task^26^ or an auditory perception task (*unpublished*) in the MEG scanner or perform an emotional recognition task outside the MEG scanner^27^. The data from these tasks are not part of NatMEG-PD, and hence these tasks are not described in further detail here.

### Clinical scales

Once all tasks were completed, participants were given a short break and subsequently proceeded to an adjacent testing room for additional clinical tests. All tests were done by neurologists or psychologists who were extensively trained in administering the tests. All clinical tests on patients were done while the patients were ON their regular dose of dopamine replacement therapy.

General cognitive ability was assessed in all participants with the Montreal Cognitive Assessment battery (MoCA)^28^, executive cognitive functions with the Fontal Assessment Battery (FAB)^29^, and the emotional state with Beck’s Depression Inventory II (BDI)^30^. A group-level summary of test scores is presented in Table 1.

Motor disease severity was characterised using PD-specific clinical scales. Motor symptoms were assessed using the Movement-Disorder Society Unified Parkinson’s Disease Rating Scale (MDS-UPDRS-III)^31^, and the disease stage was rated according to Hoehn & Yahr stage^23^.

The Levodopa equivalent daily dose (LEDD) of the patients’ regular prescribed medication was calculated according to Tomlinson *et al*.^32^ based on the patients’ medical records when these were available (for 63 of 66 patients).

### MRI data acquisition

A structural MRI was recorded on a separate day after having participated in the MEG session (no more than one month apart). The MRI data consist of 3D T1-weighted magnetisation-prepared rapid gradient-echo (MPRAGE) sequence structural images (voxel size: 1x1x1 mm) obtained on a GE Discovery 3.0 T MR scanner. The MRI was obtained for morphological analysis and for creating source spaces for MEG source reconstruction.

MRI was obtained for 121 of 134 participants (see Table 2). One participant declined to do the MRI scanning. For one participant, an MRI scanner malfunction resulted in corrupted data files. 11 participants had their scheduled MRI scans cancelled due to Covid-19-related lab restrictions in March 2020. In total, 2 PD patients and 11 healthy controls did not complete the MRI acquisition.

**Table 2:** Number of data files available per group.

	Parkinson’s patients	Healthy controls
Resting state MEG	66	68
Motor task MEG	66	68
Proprioceptive stimulation MEG	62	65
Individualised warped template MRI	64	57

### MEG data processing

The MEG was processed with MaxFilter (Elekta Oy, Helsinki, Finland) by applying temporal signal space separation (tSSS) to suppress artefacts from outside the scanner helmet and correct head movement during the recording^33^. The tSSS had a buffer length of 10 s and a cut-off correlation coefficient of 0.95. The MaxFilter procedure included correction of bad channels. Movement correction was done by shifting the head position to a position based on the median of the continuously recorded head position during the recordings for each of the three tasks separately. Empty room data was processed with the same tSSS settings but without applying movement correction.

The MEG data files were anonymised by purging subject identifiable metadata and recording date from the MEG files with the build-in anonymisation function in MNE Python^34^ and custom anonymisation scripts. Participant names in the database (sub-NNN) were assigned at random, and the file linking the new name with the original name was deleted once the BIDS conversion was complete.

No further processing was applied to the MEG data.

### MRI data processing

In line with the ethical approval, the raw MRIs are not provided to protect the privacy of the research participants. Instead, we provide individually warped templates for all participants who had structural MRIs. In short, the real MRI was first aligned to the head points measures during MEG preparation using the iterative closest points algorithm in FieldTrip^35^. A template MRI (the *Colin27* template^36^) was then warped to the individual aligned MRIs using the nonlinear spatial normalisation procedure in SPM12^37^. This gives a template MRI volume for each participant where the gross template anatomy matches the anatomy of the individual participant at a level sufficient to use the warped templates for MEG source analysis. The procedure to construct the warped MRI templates is described in detail and evaluated in Vinding & Oostenveld^38^.

### BIDS conversion

**Fig. 2:**
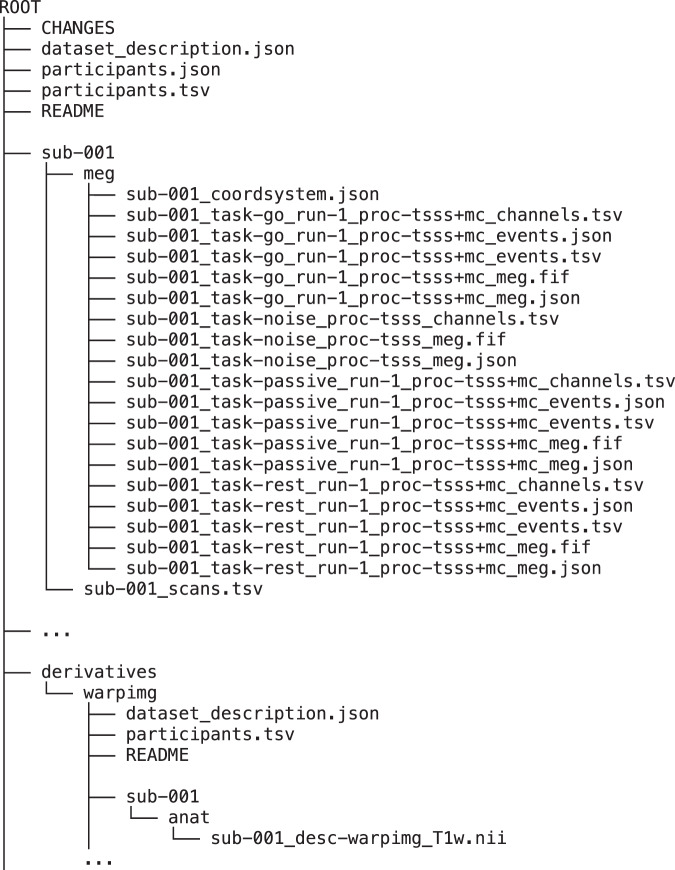
Directory and file structure of NatMEG-PD. Only the folders for the first participant are shown (sub-001); the structure is identical for all other participants.

Finally, the data were organised according to the Brain Imaging Data Structure (BIDS)^22,39^. The MEG files were organised using the *data2bids* function in FieldTrip^35^, which arranges the files according to the BIDS convention and creates the appropriate sidecar files with metadata for each MEG data file. Additional metadata (Table 3) was manually added to the data structure using custom scripts (available at https://github.com/natmegsweden/NatMEG-PD_data). Metadata, clinical test scores, and disease-related scores are provided in the *participants.tsv* file (see below). The individual warped template MRIs are provided in the derivatives folder following the BIDS convention for processed data (see *Data Records* and Fig. 2).

**Table 3:** Overview and explanation of metadata and clinical test scores included in NatMEG-PD.

Variable	Variable name	Explanation	Notes
Group	Group	Indicating whether the participants belong to the patient or control group.	
Age	Age	Age binned into five-year age bins: 45–50, 51–55, 56–60, 61–65, 66–70, 71–75, and 76–80 years.	
Sex	Sex	Biological sex. “F” for female, “M” for male.	
Handedness	Handedness	Self-reported handedness. “l” for left-handed, “r” for right-handed.	
Disease duration	disease_dur	Parkinson’s disease duration in whole years since the first diagnosis.	patients only
Levodopa equivalent daily dose	LEDD	Levodopa equivalent daily dose calculated according to Tomlinson *et al*.^32^ based on the patient’s medical records.	patients only
Becks Depression Inventory	BDI	Score on Beck’s Depression Inventory II.	
MoCA	MoCA	Score on the Montreal Cognitive Assessment.	
FAB	FAB	Score on the Frontal Assessment Battery.	
Hoehn & Yahr stage	HY_stage	Hoehn & Yahr stage.	patients only
UPDRS-III	UPDRS_III	Score on MDS-UPDRS part III.	patients only
Midline function	UPDRS_III_f1	Combined score on midline function items on MDS-UPDRS part III.	patients only
Rest tremor	UPDRS_III_f2	Combined score on rest tremor items on MDS-UPDRS part III.	patients only
Rigidity	UPDRS_III_f3	Combined score on rigidity items on MDS-UPDRS part III.	patients only
Bradykinesia right upper extremity	UPDRS_III_f4	Combined score on bradykinesia right upper extremity items on MDS-UPDRS part III.	patients only
Bradykinesia left upper extremity	UPDRS_III_f5	Combined score on bradykinesia left upper extremity items on MDS-UPDRS part III.	patients only
Postural and kinetic tremor	UPDRS_III_f6	Combined score on postural and kinetic tremor items on MDS-UPDRS part III.	patients only
Lower limb bradykinesia	UPDRS_III_f7	Combined score on lower limb bradykinesia items on MDS-UPDRS part III.	patients only

All scores are found in the participants.tsv file. The explanations are also found in the participants.json dictionary.

Data formatting compliance with the BIDS format was checked with the online BIDS validator tool (https://bids-standard.github.io/bids-validator/).

## Data Records

NatMEG-PD is available through the EBRAINS Human Data Gateway^40^. The dataset is arranged according to the BIDS standard^39^. Fig. 2 presents an outline of the folder and file structure. The repository contains folders with the MEG data for each participant (*sub-001* up to *sub-134*) with accompanying sidecar files representing the metadata. Each participant folder has a subfolder named *meg* containing the resting-state (*_task-rest_* in the filename), go task (*_task-go_* in the filename), and passive movement (*_task-passive_* in the filename) data, as well as an associated empty room recording (*_task-noise_* in the filename). The average size of the MEG data per participant is around 3.5 GB. The total size of the entire dataset is 461 GB.

There are exceptions in the file and directory structure where not all data are present, e.g., if a task condition was not recorded for a specific participant—see Table 2 for a complete overview. The data exceptions are documented in the README file at the top level of the data repository. Each participant folder contains a *_scans.tsv* file describing the MEG data files available for that participant. The data does not contain annotations of artefacts such as eye blinks or transient movements.

The MEG data in NatMEG-PD are provided as raw data, apart from the application of MaxFilter and anonymisation described above. No further data cleaning, artefact rejection, or processing has been applied to the data. The MEG data files are stored in their native FIFF (*.fif*) format. All MEG data files are accompanied by sidecar files with metadata, channel specification, event definition, and coordinate system specification, according to BIDS. The _*event.tsv* files describe the trigger codes, including the trigger onset time, data sample, trigger channel, and trigger value, which have been extracted from the trigger channel in the corresponding MEG data file. The *event_type* column contains descriptions of which event the trigger values represent. Exceptions to trigger values are documented in the dataset README file.

NatMEG-PD does not contain raw MRI data. To ensure the anonymity of the research participants, we provide individually warped templates (see *MRI data processing*). The folder *derivatives/warpimg/* contains the individualised warped template MRIs for each participant with an original MRI (see Table 2). The individualised warped templates are available in NIfTI (*.nii*) format and found in the *anat* subfolder within each subject folder (see Fig. 2). The dataset description file in the *warpimg* folder has a link to the GitHub repository with the code used to create the individualised warped templates (see *Code Availability*).

Metadata about the participants is located at the top level of the data repository in the *participants.tsv* file, with the data dictionary in the *participants.json*. Each row in the *tsv* file represents information about one participant, including which group the participant belongs to (PD patient or healthy control), age, sex, and handedness, as well as the test scores on the MoCA, BDI, and FAB for all participants. For the PD patients, it further contains disease duration, Levodopa equivalent daily dose (LEDD), Hoehn & Yahr stage, and score on the MDS-UPDRS-III (Table 3). In addition to the total MDS-UPDRS-III scores, the metadata also contains symptom-specific subscales of MDS-UPDRS-III based on the factors described by Goetz *et al*.^41^. The subscales are *midline function*, *rest tremor*, *rigidity on right-side, rigidity on left-side*, *upper-body bradykinesia*, *postural and kinetic tremor*, and *lower limb bradykinesia*.

## Technical Validation

### MEG

The data quality of the MEG data was monitored through all recordings. The signal quality of all MEG channels was visually inspected prior to each recording. Channels with visually distinct noise or repeated “jumps” were heated to remove trapped flux and, thereby, noise—after which channels were again inspected. This procedure was repeated until the data was deemed at a sufficient quality by the experimenters. The participant’s head position inside the MEG scanner was assessed first in the MSR by asking if they felt the top and back of their head against the helmet. Second, the head position was inspected outside the MSR through visualisation of the head position based on the measured HPI locations. This check was performed before each task began, and if it indicated that the participant had shifted position, the experimenters would reposition the participant accordingly.

The quality of the EOG channels was assessed prior to the start of the session by having the participant blink three times and confirm that clear blink activity was visible on the EOG channels. The quality of the ECG data was similarly assessed by confirming that the ECG data had visible QRS- and T-waves. The accelerometer data was checked by asking the participants to move their hand.

Adherence to the task instructions was always checked before the MEG recordings began for each task. In the resting state recording, the experimenters ensured that the participants had closed their eyes using the video of the participant before pressing a key that would start the timer for the three-minute resting-state period. The start is signified by a trigger in the MEG data, and after three minutes, a second trigger was added to the MEG data to signify the end of the task. Five resting state recordings are missing either start or stop triggers. In those cases, we recommend using either the start of the file, or three minutes after the start trigger, respectively. All exceptions to triggers are documented in the BIDS README file.

For the go task, participants were first given training trials to familiarise themselves with the task. Once the experimenters had ensured that the participant understood the task and performed the task correctly, the recording began. Before the passive movements, the experimenters ensured that the right index finger was located on the passive movement stimulator. The participant received a few stimulations before the recording began, and the movements were confirmed by monitoring the accelerometer signals.

**Fig. 3:**
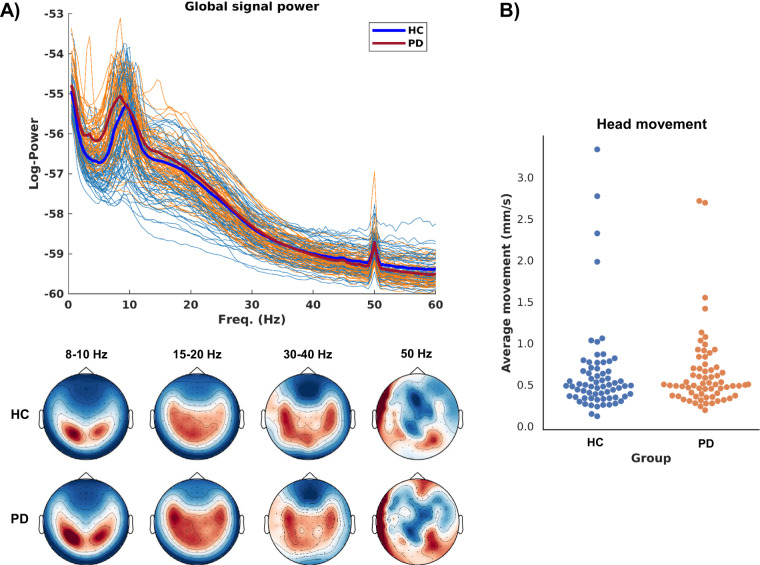
(**A**) The spectral power of the entire raw resting-state data for all participants. Each line is the PSD for a single participant colour coded by group (orange for patients, light blue for healthy control). The thick lines are group means (red for patients, dark blue for healthy controls). The topography of the group averaged power is shown below for the frequency bands 8-10 Hz (alpha), 15-20 Hz (low beta), 30-40 Hz (low gamma), and 50 Hz (line noise frequency). (**B**) Summary of the average head movement during the resting state recording per participant split between groups.

The MEG data is provided as raw data. Apart from MaxFilter cleaning with movement compensation and purging metadata for anonymisation, no further data cleaning, artefact rejection, or processing has been applied. The data does, therefore, not contain annotation of artefacts such as eye blinks or transient movements. The quality assessment of MEG data included the online inspection during data acquisition and post hoc inspection of signal power (Fig. 3A), and group-level comparison of the participants’ head movement measured by the HPI inside the MEG helmet (Fig. 3B). There was no significant group-level difference in the average head movement between healthy controls and PD patients (Mann-Whitney *U* = 2051, k = 0.47; median HC: 0.49 mm/s, median PD: 0.50 mm/s). The recorded continuous head movements are stored in the MEG data files as quaternions in the channels CHPI001-009, which can be read with MEG analysis software, and researchers can mark and reject segments with movements if they wish so.

The rationale for providing raw data is that the optimal procedure for MEG data cleaning may vary from application to application. The raw data can accommodate different needs for data cleaning and analyses.

### MRI

The quality of the T1 weighted images was inspected at the recording site. For one recording, we experienced a scanner malfunction which, for unknown reasons, led to a corrupted file; this was not discovered until offline processing of the MRI data. This data is marked as missing data and documented in the NatMEG-PD README file. All warped templates were inspected manually to ensure the warping procedure had completed without error. The quality of the individualised warped templates was inspected manually, first by visually inspecting that the warped templates did not have gross warping errors and second, by visually comparing the gross anatomy of the head shape of the warped template and original MRI by overlaying the images.

## Usage Notes

In accordance with the General Data Protection Regulation (GDPR), the data in NatMEG-PD is available through the EBRAINS Human Data Gateway^40^. Access to the dataset must be requested; this requires the user to have an EBRAINS account for authentication. Access is granted if the EBRAINS Data Usage Agreement for human data is explicitly accepted by the authenticated user. User accounts are available to everyone with a legitimate interest. The EBRAINS Access Policy requires all users to create an account and identify themselves using an institutional email address. Users without an institutional email address can request an account by contacting EBRAINS and provide a short motivation on why they need access (for precise information, see https://www.ebrains.eu/page/sign-up).

### MEG data

The data in NatMEG-PD was obtained for two separate research projects. The resting-state data were obtained for analysis of beta burst. The motivation and results are described by Vinding *et al*.^42^. The aim of the go task and passive movement task was to examine the beta ERS and ERD in PD patients and healthy (similar to^8^). However, the nature of MEG data and the tasks means many potential features in the data can be extracted from this dataset.

Resting state MEG can, for example, be analysed in terms of its spectral content by spectrally decomposing the signals. Spectral analysis might focus on specific frequency bands—for example, changes in resting state beta band have often been linked to PD—or analyses that investigate broad-band features of the signal. These analyses can be confined to local activity—e.g., by focusing on sensorimotor signals—or can investigate features across the entire montage to give a picture of either the global changes (e.g., by calculating the global mean field) or by looking for specific spatial changes across areas of the brain. The temporal and spatial resolution of MEG also allows for functional connectivity analysis by calculating one (or more) connectivity measures between the signals, again, either by focusing on specific connections or one-to-all connectivity that, for example, can be summarised by graph-theoretical metrics.

The two movement tasks are ideal for investigating evoked responses in the time domain or induced responses in the time-frequency domain time-locked to the movements. This is done by segmenting the data relative to the movement onset (marked by triggers in the data) and averaging across the segments or time-frequency representation of the segments. The movement tasks were designed to elicit the canonical movement-related beta band response seen around the sensorimotor cortex, which shows a pre-movement desynchronisation followed by a post-movement increase in synchronisation. In addition to the local movement-related beta-band dynamics, it is also possible to explore other frequency bands of interest. It is also possible to expand the analysis to whole-head activity by including the entire MEG sensor array or explore connectivity between specific regions of interest or extract connectivity metrics for the entire brain.

The MEG data is provided as raw data without any pre- or post-processing applied, except for the MaxFilter procedure and anonymisation described above. It can, therefore, be used for a wide range of MEG analysis that covers the scope of most MEG analyses prevalent today. We choose to include the raw MEG data at the stage prior to any data cleaning or removal of artefacts to increase the reusability and utility of the data, compared to releasing the data processed according to the specifications used for our analysis. The criteria for data cleaning and artefact rejection differ depending on which feature in the MEG data one is interested in. Different research groups have different preferences on how to process neural time-series data^43^. Raw data requires that researchers apply their own quality assessment of the data and implement data cleaning as they find necessary. We strongly encourage researchers to ensure that the data quality confirms to their standards for the analyses they wish to apply. The data is provided as raw, which means that typical MEG artefacts—such as eye blinks or heartbeats—have not been removed from the data. All task data is also provided with all trials as collected, meaning that both correct responses and trials with errors are present in the data. Reaction times can be calculated from the triggers in the data or the movement onset from the accelerometer, so researchers can use their own criterion for including/discarding trials. The data cleaning and pre-processing procedures we used for the resting state data are described in Vinding *et al*.^42^ that can serve as inspiration for re-analysis.

The MEG data files contain the recorded H/VEOG and ECG channels, which are commonly used to guide MEG data cleaning. If researchers want to apply more advanced analyses to the EOG and ECG data, we strongly advise that they check the data quality, as the EOG and ECG were acquired only for MEG artefact correction and, therefore, might not be as carefully monitored as if they were collected for dedicated EOG or ECG analysis.

The MEG data files are provided in the native FIFF format, which can be read by all MEG data analysis software and toolboxes. Each MEG data file is accompanied by corresponding sidecar files with metadata for the MEG data files, channel specification, event definition, and coordinate system specification, as well as describing trigger onset time, data sample, trigger channel, and trigger value. Note that because the MEG system had multiple trigger channels, the same event appears several times in different channels. The triggers named STI001-STI016 contain binary trigger pulses for each pin, and the trigger channel STI101 contains the composite values. For most purposes, it is sufficient and recommended to use the STI101 trigger channel. Exceptions to trigger values are documented in the dataset README file.

Movements in the go task and passive movement task are marked by separate triggers. Reaction times in the go task can be calculated from the movement triggers relative to the go cue trigger. Additionally, finger movement kinematics can be evaluated using the accelerometer data. The accelerometer data is represented by three channels measuring the three orthogonal axes—labelled MISC13–15 in the data files. The axes are relative to the direction of the accelerometer attached to the tip of the index finger of the participants, and the units of the accelerometer channels are in arbitrary units. It is recommended to calculate the absolute acceleration by taking the Euclidian mean of the three accelerometer channels when analysing the movement kinematics. The accelerometer data is useful for extracting precise movement onset times, duration of movements, and potentially other features from the movement kinematics (for examples see^44^). The accelerometer data is present in all three MEG task files (not just the movement-related tasks) and can be used to check for movement kinematics in the rest data or before and after movements in the movement-related tasks (e.g., to discard trials with movements in the pre-stimulus time window).

Contained in the MEG FIFF files are the fiducial nasion-, left-, and right pre-auricular points and head points measured with the Polhemus Fastrak tracker during MEG preparations. Most MEG analysis software can read the head points from the files.

### MEG source analysis

Analysis of MEG (both resting-state and event-related) can be done in the native sensor space or in source space by applying source reconstruction methods to model the neural origin of the measured signals. The relatively high spatial resolution of MEG makes it possible to estimate and separate the contribution of different brain areas. Source reconstruction methods require a model of the source space and a forward model describing how signals from the sources reach the sensors. Source models and forward models are usually derived from anatomical MRI using the inner skull boundary for each participant for the highest precision in the source reconstruction^45,46^. The individualised warped template preserves the gross shape of the head and inner skull boundary (needed for MEG source reconstruction) at the individual level but anonymises the anatomical fingerprint of the MRI. For analysis that uses source reconstruction to unmix signals or focus reconstructed time series, the warped templates should provide comparable results to the original MRI.

It should be noted that the warped templates might not be suited for all types of MEG source analysis, e.g., if one is interested in locating the exact point that is the anatomical origin of a signal within individual subjects, as the brain anatomy is altered in the warped templates. However, analyses that aggregate over anatomical labels or focus on a patch region of interest, e.g., based on anatomical atlases, do produce comparable results to similar analyses performed on the participant’s original MRI^38^. Most analyses that do not focus on locating an exact anatomical point at the individual level should be possible with the provided data. We cannot definitively answer whether the individualised warped template MRIs are adequate for any given research question or MEG source reconstruction method. We encourage researchers to use their expert knowledge about how their desired source reconstruction method works to judge for themselves whether the individualised warped templates are appropriate to use with their source reconstruction method of choice.

The individualised warped template MRI files are provided in standard NIFTI (*.nii*) format that can be read as any other MRI by most software and toolboxes used for MEG source analysis. The 13 participants without MRI (see *MRI data acquisition*) do not have individualised warped template MRI. For these data, researchers will have to find alternative ways to construct anatomical models for source analysis if included in source analysis, e.g., by scaling an MRI template to match the head surface points that are recorded with the Polhemus tracker (found in the MEG data files) or to scale the template to match the individual participant’s head circumference^47^—or simply not include these in the source analysis.

### Note on individualised warped template MRI

Note that NatMEG-PD does not contain raw MRI data. To ensure the anonymity of the research participants, we provide individually warped templates for all participants who had structural MRIs. The individually warped effectively alter the structural “fingerprints” of the individual MRIs^38^. The MRIs in NatMEG-PD are provided with the intention of facilitating MEG source analysis but are not recommended for structural analysis.

### Clinical test scores

NatMEG-PD contains clinical test scores (see Table 3) to characterise the participants along different dimensions: overall cognitive ability (MoCA), executive functions (FAB), and signs of depression (BDI). The PD patient group is further characterised by clinical information, including the assessment of motor symptoms (MDS-UPDRS-III), disease stage (Hoehn & Yahr stage), as well as disease duration and LEDD. In addition to the overall score on MDS-UPDRS-III, the score was divided into subscales related to more specific motor symptoms (see Table 3). The test scores are provided in an easy-to-read table format in the *participants.tsv* file. These can either be imported and used as dependent or independent variables in the analysis of MEG features or used as covariates in the analysis. We hope that this can facilitate the exploration of how specific motor symptoms are related to or can be predicted by functional neural activity.

The clinical test measures represent a simple but informative characterisation of the disease phenotype of the patients. The included test scores represent the international clinical standards for characterising motor symptoms, cognition, and emotion in PD, which makes it easy to compare across studies and generalise results.

## Data Availability

The scripts used to anonymise and arrange the data according to BIDS format are available at https://github.com/natmegsweden/NatMEG-PD_data. The scripts for the MRI warping procedure are available from https://github.com/mcvinding/warpimg.
